# 

**Published:** 1905-06

**Authors:** 


*The following donations have been received since the publication of the List in March:*

*May 31st, 1905.*

| | |
|---|---|
| British Balneological and Climatological Society (1) | 1 volume. |
| Richard Caton, M.D. (2) ... ... ... ... ... ... ... | 1 ,, |
| L. M. Griffiths (3) ... ... ... ... ... ... ... ... | 4 volumes. |
| The Middlesex Hospital (4) ... ... ... ... ... ... | 1 volume. |
| Rhode Island Medical Society (5) ... ... ... ... | 1 ,, |
| R. Shingleton Smith, M.D. (6) ... ... ... ... ... | 4 volumes. |
| James Swain, M.D. (7) ... ... ... ... ... ... ... | 2 ,, |

Unbound periodicals have been received from Mr. J. Dacre and Dr. R. Shingleton Smith. Mr. L. M. Griffiths has presented some unframed portraits and pamphlets.

### FIFTY-SIXTH LIST OF BOOKS.

The titles of books mentioned in previous lists are not repeated.

The figures in brackets refer to the figures after the names of the donors, and show by whom the volumes were presented. The books to which no such figures are attached have either been bought from the Library Fund or received through the *Journal.*

| | | | |
|---|---|---|---|
| **Allbutt, T. C.** | *The Historical Relations of Medicine and Surgery* | | 1905 |
| **Barclay, A. W.** | *On Gout and Rheumatism* | (3) | 1866 |
| **Barrett, A. W.** | *Dental Surgery* | 4th Ed. | 1905 |
| **Bennett, Sir W.** | *Recurrent Effusion into the Knee-Joint* | | 1905 |
| **Birt, E.** | *Notes on Clinical Blood Pressure Instruments* | | 1904 |
| **Blair, C.** | *Errors of Refraction* | | 1905 |
| **Burnet, R. W.** | *Foods and Dietaries* | 4th Ed. | 1905 |
| **Caton, R.** | *The Harveian Oration* | (2) | 1904 |
| **Froussard, P.** | *Mucomembranous Enterocolitis* (Ed. by E. Blake) | | 1905 |
| **Griffiths, L. M.** | *Medical Philology,* Part I. | | 1905 |
| **Hall and G. Herxheimer, I. W.** | *Methods of Morbid Histology and Clinical Pathology* | | 1905 |
| **Hall, M.** | *Observations in Medicine* | (3) | 1845 |
| **Herxheimer, I. W. Hall and G.** | *Methods of Morbid Histology and Clinical Pathology* | | 1905 |
| **Jellett, H.** | *A Manual of Midwifery* | | 1905 |
| **Lack, L. A. H.** | *An Introduction to Physiology* | | 1905 |
| **Lake, R.** | *Laryngeal Phthisis,* 2nd Ed. (Ed. by H. Barwell) | | 1905 |
| **Lane, W. A.** | *Operative Treatment of Chronic Constipation* | (6) | 1904 |
| **Leaf, C. H.** | *Common Diseases of the Rectum and Anus* | | 1905 |
| **Lucas, E. W.** | *The Book of Prescriptions* (Beasley) | 8th Ed. | 1905 |
| **Maylard, A. E.** | *Abdominal Pain* | | 1905 |
| **Miller, W. A.** | *Elements of Chemistry* (6) Part I., 2nd Ed. 1860; II., III., | | 1856–62 |
| **Mingazzini and G. Perusini, G.** | *Heredo-Spinal Atrophy* | | 1904 |
| **Moorhead, T. G.** | *Surface Anatomy* | | 1905 |
| **Palmer, C. F.** | *Inebriety: its Source, Prevention, and Cure* | | [N.D.] |
| *Pamphlets,* 3 vols. | | | [V.Y.] |
| **Perusini, G. Mingazzini and G.** | *Heredo-Spinal Atrophy* | | 1904 |
| **Plumbe, S.** | *On the Value of Vaccination in Small-pox* | (3) | 1832 |
| **Smith, L.** | *Golden Rules of Medical Practice* | 6th Ed. | [1905] |
| **Stuart-Low, W.** | *Mucous Membranes* | | 1905 |
| **Symes, J. O.** | *The Rheumatic Diseases* | | 1905 |
| **Taylor, A.** | *The Sanitary Inspector's Handbook* | 4th Ed. | 1905 |
| **Thomson, S.** | *Health Resorts in Britain* | (3) | 1860 |
| **Turner, G. R.** | *Clinical Lectures on Appendicitis, Hernia, & Gastric Ulcer* | | 1905 |
| **Vallack, A. S.** | *Asepsis* | | 1905 |
| **Wallace, J. S.** | *Modern Dietetics in the Causation of Disease* | | 1905 |

### TRANSACTIONS, REPORTS, JOURNALS, &c.

| | | |
|---|---|---|
| Albany Medical Annals | Vol. XXV. | 1904 |
| Alienist and Neurologist, The | Vol. XXV. | 1904 |

American Dermatological Association, Transactions of the ... ... 1904
American Journal of Obstetrics ... ... ... ... ... ... ... ... Vol. L. 1904
American Medicine ... ... ... ... ... ... ... ... ... ... ... Vol. VIII. 1904
American Practitioner and News, The Vols. XXXVII., XXXVIII. 1904
Archives de Neurologie ... ... ... ... ... ... Tom. XVII., XVIII. 1904
Archives of Pediatrics ... ... ... ... ... ... ... ... ... Vol. XXI. 1904
Archives of the Middlesex Hospital ... ... ... ... ... ... (4) Vol. V. 1905
Australasian Medical Gazette, The ... ... ... ... ... ... Vol. XXIII. 1904
Birmingham Medical Review, The ... ... ... ... ... ... ... ... ... 1904
Bookseller, The... ... ... ... ... ... ... ... ... ... ... ... ... ... 1904
Boston Medical and Surgical Journal, The ... ... ... Vol. CLI. 1904
British Journal of Children's Diseases ... ... ... ... ... Vol. I. 1904
British Journal of Dermatology, The ... ... ... ... Vol. XVI. 1904
Brooklyn Medical Journal, The ... ... ... ... ... ... ... Vol. XVIII. 1904
Bulletin de l'Académie de Médecine ... ... ... ... Tom. LI., LII. 1904
Bulletin de l'Institut international de Bibliographie ... ... ... 1902, 1903
Caledonian Medical Journal, The ... ... ... ... N.S., Vol. V. 1904
Canadian Practitioner and Review, The ... ... ... ... Vol. XXIX. 1904
Cincinnati Lancet-Clinic, The ... ... ... ... ... ... Vol. XCII. 1904
College of Physicians of Philadelphia, Transactions of the Vol. XXVI. 1904
Congrès international de Medécine (xiv^e^), Comptes Rendus de Section de Chirurgie ... ... ... ... ... ... ... ... ... ... ... (7) 1904
English Catalogue of Books, The ... ... ... ... ... ... ... ... ... 1905
Gazette des Hopitaux de Toulouse ... ... ... ... ... ... ... ... ... 1904
Gazette médicale de Paris ... ... ... ... ... ... 13^e^ Sér., Tom. IV. 1904
Gazzetta Medica Lombarda ... ... ... ... ... ... ... ... ... ... ... 1904
Giornale internazionale delle Scienze mediche ... ... ... ... ... 1904
Glasgow Medical Journal, The ... ... ... ... ... ... Vol. LXII. 1904
Henry Phipps Institute for the Study of Tuberculosis, First Annual Report of the ... ... ... ... ... ... ... ... ... ... ... 1903-04
Hospitals and Charities, Burdett's ... ... ... ... ... ... ... ... 1905
Indian Medical Gazette, The ... ... ... ... ... ... Vol. XXXIX. 1904
Journal of Balneology and Climatology ... ... ... (1) Vol. VIII. 1904
Journal of Hygiene, The ... ... ... ... ... ... ... ... ... Vol. IV. 1904
Journal of Laryngology, The ... ... ... ... ... ... ... Vol. XIX. 1904
Journal of Medical Research, The ... ... ... ... ... Vol. XII. 1904
Journal of Mental Science, The ... ... ... ... ... ... ... Vol. L. 1904
Journal of Nervous and Mental Diseases, The Vols. XXX., XXXI. 1903-04
Journal of Obstetrics and Gynæcology of the British Empire, The Vol. VI. 1904
Laboratory Reports, Royal College of Physicians of Edinburgh. Vol. IX. 1905
Library Association Record, The ... ... ... ... ... Vol. VI. 1904
Library Association Year Book ... ... ... ... ... ... ... ... ... 1905
Library, The ... ... ... ... ... ... ... ... ... N.S., Vol. V. 1904
Medical Age, The ... ... ... ... ... ... ... ... ... ... Vol. XXII. 1904
Medical Annual, The ... ... ... ... ... ... ... ... ... ... ... ... 1905
Medical Chronicle, The ... ... ... ... ... ... ... N.S., Vol. VII. 1904
Medical Press and Circular, The ... ... ... ... [Vol. CXXIX.] 1904
Medical Record, The ... ... ... ... ... ... ... ... ... Vol. LXVI. 1904

| | | |
|---|---|---|
| Medical Review, The ... ... ... ... ... ... ... ... | Vol. VII. | 1904 |
| Medical Society's Transactions ... ... ... ... ... ... | Vol. XXVII. | 1905 |
| Mercy and Truth ... ... .. ... ... ... ... ... ... | Vol. VIII. | 1904 |
| Monthly Cyclopædia of Practical Medicine, The ... ... | Vol. VII. | 1904 |
| Montreal Medical Journal, The ... ... ... ... ... | Vol. XXXIII. | [1904] |
| Nord médical, Le ... ... ... ... ... ... ... ... ... | Tom. X. | 1904 |
| Northumberland and Durham Medical Journal, The ... ... ... ... | | 1904 |
| Post-Graduate, The ... ... ... ... ... ... ... ... ... | Vol. XIX. | 1904 |
| Progrès médical, Le... ... ... ... ... ... | 3e Sér, Tom. XIX., XX. | 1904 |
| Progressive Medicine .. ... ... ... ... ... ... ... ... | Vol. I. | 1905 |
| Revue générale d'Ophtalmologie ... ... ... ... ... ... | Tom. XXII. | 1904 |
| Rhode Island Medical Society, Transactions of the (5), | ol. VII., Pt. I. | 1904 |
| Scottish Medical and Surgical Journal, The ... ... ... | Vol. XV. | 1904 |
| Therapeutic Gazette, The ... ... .. ... ... ... | Vol. XXVIII. | 1904 |
| Travaux d'Obstétrique et de Gynécologie ... ... ... ... | [Vol. XX.] | 1904 |
| Wiener klinische Wochenschrift ... ... ... ... ... ... ... ... ... | | 1904 |
| Zentralblatt für Chirurgie ... ... ... ... ... ... . ... ... ... | | 1904 |
| Zentralblatt für Gynäkologie ... ... ... .. ... ... ... ... ... ... | | 1904 |
| Zentralblatt für innere Medicin ... ... ... ... ... ... ... ... ... | | 1904 |

### MEETINGS OF SOCIETIES.

### Bristol Medico-Chirurgical Society.

### Local Medical Notes.

**University College, Bristol.**—Newman Neild, M.B., B.Ch. Vict., has been appointed Lecturer on Pharmacology and Therapeutics, and O. C. M. Davis, B.Sc. Lond., Lecturer on Materia Medica and Practical Pharmacy, at the College.

Students of the College have been successful in the following examinations:—

University of London, M.B., B.S.—A. Coleridge, C. A. Moore.

Conjoint Board of England.—First Examination, *Chemistry and Physics:* R. C. Clarke, J. H. Morgan, A. J. O. Wigmore. *Elementary Biology:* C. H. Hart, H. B. Logan, F. C. Morgan, W. A. Reynolds, T. S. Rippon. *Practical Pharmacy:* A. H. C. Dawes, R. G. Vaughan. *Chemistry only:* D. M. Cox. Second Examination, *Anatomy and Physiology:* J. M. Hammond. Final Examination, *Medicine:* H. O. Gough, J. E. Jones, A. N. Thomas. *Surgery:* J. S. Avery, R. C. Bright, V. A. Crinks.* *Midwifery:* J. H. Board, A. J. M. Wright.

Society of Apothecaries.—*Medicine:* J. E. Jones.*

**Conversazione.**—On Friday evening, May 19th, a conversazione was given by University College, and was attended by 1,200 guests. The buildings lost their every-day appearance under an effective arrangement of bunting and banners, and the large number of stewards helped to make the arrangements very

* Completes Examination.

satisfactory. A reception was held in the large hall by the Chairman of the Council (the Right Hon. Lewis Fry), the Principal (Professor C. Lloyd Morgan) and the Dean of the Faculty of Medicine (Professor E. Markham Skerritt), after which the guests separated to various parts of the building. In the library of the Faculty of Arts Mr. Bligh Bond showed his harmonograph, where were also given X-ray demonstrations. The vortex rings in the geological lecture rooms were very interesting, as were also the various experiments in the physics department. In the physical laboratory one could handle radium and see experiments on the dispersal of fog. In the chemical laboratory many experiments were shown, but the demonstration on liquid air attracted a large number of people, as did also the bacteriological exhibits. In the medical school, which was barely recognisable with its fairy lights and other paraphernalia, many things of interest were shown, including high frequency and high tension electric currents, fatigue of muscle, pulse tracings, personal reaction time, beating heart of tortoise, section-cutting instruments, experiments on colour, spectra of blood, and finger-prints. The medical library was used for refreshments.

**The Long Fox Lecture** this year will be delivered in November by Dr. Markham Skerritt. The subject of the lecture and exact date will be announced later.

**Bristol Royal Infirmary.**—John Roger Charles, M.A., M.D. (Cantab.), M.R.C.P.(Lond.), B.C., M.R.C.S., has been appointed Assistant Physician. Hon. Assistant Anæsthetist — S. V. Stock, M.B., B.S. Lond. Dental Anæsthetist—L. A. Moore, M.R.C.S., L.R.C.P. Resident Obstetric Officer — G. B. McCaul, M.B. Casualty Officer—A. L. Sheppard, M.B. Junior House Surgeon—F. H. Rudge, M.R.C.S., L.R.C.P.

**Bristol General Hospital.**—Senior House Surgeon—A. C. A. Van Buren, M.D., B.S. Lond. House Physician—A. R. Short, M.D., B.S., B.Sc. Assistant House Surgeon—L. N. Morris, M.R.C.S., L.R.C.P. Casualty House Surgeon—A. W. C. Richardson, M.B. Lond.

**Bristol Dispensary.**—H. R. C. Newman, M.B.Durh., M.R.C.S., L.R.C.P., has been appointed Surgeon to this institution.

**Bristol Lunatic Asylum.**—The Medical Superintendent of the Asylum (Dr. J. V. Blatchford) in his annual report to the Visiting Committee of this institution for 1904 states that the percentage of recoveries on the admissions, excluding transfer cases, was 42.59 ; 112 patients were discharged recovered during the year. There were 110 deaths, the death-rate on the average number of resident patients being 12.02 per cent.

**Bristol Eye Dispensary.**—During 1904 the patients attending this Dispensary numbered 2,315, compared with 2,268 in 1903. The financial statement showed that a small favourable balance remained at the end of the year.

**Bristol Hospital Sunday Fund.**—At a meeting of the Council of this fund, held on May 18th, it was reported that in 1905 there had been collections at 425 places of worship; of this number 55 were new ones. The total amount received was £2,053, being £326 in excess of the amount collected in 1904, and £280 more than the largest sum hitherto realised. Of this sum the Bristol Royal Infirmary has received £805, the General Hospital £675, the Children's Hospital £261, and the Eye Hospital £120; £108 were divided among smaller medical charities, giving a total of £1,969 which has been already distributed.

**Newport Infirmary.**—V. A. Crinks, M.R.C.S., L.R.C.P., has been appointed House Physician.

**Staffordshire General Infirmary.**—Arthur Bernard Cridland, F.R.C.S. Edin., M.R.C.S. Eng., L.R.C.P. Lond., has been appointed Honorary Ophthalmic Surgeon.

**Isolation Hospitals.**—On April 8th one of the provincial meetings of the Royal Sanitary Institute was held at Bristol, when the Medical Officer of Health (Dr. D. S. Davies) opened a discussion on isolation hospitals. The City Engineer gave an interesting account of the Bristol Isolation Hospitals. Considering the population of the town (358,000), some speakers thought that the number of available beds (196) was inadequate.

### BATH.

**Public Health**—The Medical Officer of Health of the City of Bath (Dr. W. H. Symons) states in his annual report that the corrected death-rate during 1904 was 13.3 per 1000, and the infantile mortality rate 111 per 1000 births. Although the deaths among young children are fewer than in many parts of England, Dr. Symons thinks that a still greater reduction might be made, and refers to the good work which is being done elsewhere by female inspectors, one of whom might be usefully employed in Bath. There were forty-five deaths from pulmonary tuberculosis, a disease which is voluntarily notifiable in Bath, seventy-six cases thus coming to the notice of the Medical Officer of Health during the year.

### EXETER.

**Royal Devon and Exeter Hospital.**—A few months ago Mrs. Nosworthy, of Dawlish, gave the sum of £1,000 for the purpose of erecting a new operating theatre for the Royal Devon and Exeter Hospital. It has since then been discovered that the cost of the undertaking will be £1,500, and when Mrs. Nosworthy was acquainted with the fact she generously decided to give an additional £500 to complete the work.

